# Circadian Rhythm Disorders and Corresponding Functional Brain Abnormalities in Young Female Nurses: A Preliminary Study

**DOI:** 10.3389/fneur.2021.664610

**Published:** 2021-04-30

**Authors:** Xiaoli Wu, Fan Bai, Yunlei Wang, Lu Zhang, Lixu Liu, Yudong Chen, Hanzhi Li, Tong Zhang

**Affiliations:** ^1^Department of Neurorehabilitation, Rehabilitation Medicine of Capital Medical University, China Rehabilitation Research Centre, Beijing, China; ^2^China Rehabilitation Science Institute of China Rehabilitation Research Centre, Beijing, China; ^3^Beijing Key Laboratory of Neural Injury and Rehabilitation, Beijing, China

**Keywords:** melatonin, circadian rhythm, resting functional magnetic resonance imaging, regional homogeneity, functional connectivity

## Abstract

**Objective:** Shift work is associated with a decrease in melatonin level and perturbation of the circadian rhythm; however, it is unknown if these lead to functional brain changes. In this study, we investigated whether circadian rhythm disorders caused by shift work are related to changes in brain functional connectivity (FC) and regional homogeneity (ReHo) using whole-brain resting-state functional magnetic resonance imaging (fMRI).

**Methods:** This prospective case-control study included nine female night shift nurses and nine age-matched female day work nurses with normal sleep rhythms. To assess sleep quality and mood, participants were asked to complete questionnaires. Serum melatonin and cortisol levels were measured. ReHo of whole-brain resting-state function and seed-based FC of the bilateral hypothalamus were compared between groups. Variables that differed significantly between groups were used to examine the association between questionnaire scores and hormone levels and fMRI data.

**Results:** The night shift nurses had significantly lower sleep quality and melatonin levels; lower ReHo activation in the bilateral cerebellar hemisphere and higher ReHo in the bilateral occipital lobe and left parietal lobe; and higher FC from the hypothalamus to the right cingulate gyrus, right putamen, and vermis than did the day shift nurses. Activation of the right cerebellar hemisphere left superior parietal gyrus, and the right superior occipital gyrus was correlated with sleep quality scores. Moreover, activation of the right cerebellar hemisphere (*r* = 0.583, *P* = 0.011) was correlated with melatonin levels, and higher sleepiness scores were associated with stronger FC between the hypothalamus and vermis (*r* = 0.501, *P* = 0.034).

**Conclusions:** Circadian rhythm disorder caused by night shift work can lead to a decrease in sleep quality and melatonin level, as well as a series of changes in brain FC and ReHo.

## Introduction

The conflict between the social demands of modern life and endogenous rhythms has resulted in circadian rhythm disorders and a host of mental and physical health issues. These issues are typified by shift work, which is underscored by an imbalance between social work/rest time and internal circadian rhythms. With industrialization, travel across time zones, use of electronic products, and light exposure of modern long-term indoor life, individuals are forced to ignore or inhibit the natural circadian rhythm. For example, the use of personal electronic devices rich in blue light at night can delay circadian rhythm and the start of sleep, inhibit melatonin secretion, and increase morning sleepiness (1). In addition, individual sensitivity to light circadian rhythm also demonstrates individual differences. Therefore, a more in-depth understanding of circadian rhythm disorders, as well as possible hormonal changes and brain function changes, will facilitate health management and treatment.

Effects of sleep and circadian rhythm disorders on health and cognitive ability are well-documented. Indeed, prolonged night shift work is associated with an increased risk of cardiovascular disease (2). Shift work is associated with increased secretion of cortisol and catecholamines (3, 4) and decreased secretion of melatonin (5), which may result in abnormal hormone secretion rhythms. A decline in melatonin levels in the morning is related to activation of the prefrontal cortex, indicating that changes in melatonin levels in the morning may be related to cortical alertness, attention, and executive ability (6). Furthermore, a decrease in attention and alertness as well as significant changes in brain function, occur after sleep deprivation (7). Additionally, insomnia and anxiety increase significantly, and cerebral perfusion changes occur among shift workers (8). This previous evidence demonstrates that the abnormality of circadian rhythm could not only lead to hormone secretion disorders, attention deficits, and sleep disorders but also affect brain function. Differences in circadian phenotype between “owls” and “larks” are associated with differences in brain functional connectivity (FC), especially in the default mode network (DMN), which is related to attention and subjective sleep disorders (9). One study compared seed connectivity of the suprachiasmatic nucleus using resting-state fMRI to determine the possible mechanisms of circadian rhythm disorders in patients with delirium and revealed that FC from the suprachiasmatic nucleus to the dorsal anterior cingulate cortex increased, whereas FC to the posterior cingulate gyrus, parahippocampal gyrus, cerebellum, and thalamus decreased (10). These findings suggest that resting-state fMRI is a useful tool for investigating sleep disorder-associated functional changes in the brain. In addition, the resting-state fMRI studies of circadian rhythm disorder typically involve patients with sleep disorders or sleep deprivation. There is currently no research on brain function changes among healthy participants with purely circadian rhythm disorder.

Melatonin secretion is modulated by various factors; among them, light, which inhibits its secretion, has the strongest effect (11, 12). Using differential blood oxygenation level-dependent (BOLD) functional magnetic resonance imaging (fMRI), Vimal et al. found that the activation of the human suprachiasmatic area across times of day matched the known rhythm in the responsiveness of the circadian system to light (13). McGlashan et al. further found a positive correlation between the activation of the human suprachiasmatic area in response to light and melatonin suppression using BOLD fMRI (14). The suprachiasmatic nucleus is an anterior hypothalamic nucleus constituting the central system for regulating circadian rhythm in mammals. It projects strongly to the paraventricular nucleus (PVN), thalamus, lateral hypothalamus, dorsal medial hypothalamus, striatum, and supraventricular area. Hypothalamic nuclei, particularly the suprachiasmatic nucleus, regulates biological rhythms of the body, including body temperature, sleep/wake rhythm, hormones, metabolism, reproduction (11, 15), and melatonin secretion. Studies also have shown that the maintenance of sleep and awakening and conversion from one state to another are effectuated by a variety of neurons scattered in multiple brain regions. These neurons coordinate with and inhibit each other to dynamically regulate sleep (16–18). The hypothalamus is thought to be the control center of circadian rhythm (19). A study assessing the relationship between hypothalamic volume and sleep disorders reported significant decreases in sleep efficiency and volume of hypothalamic gray matter in patients with Huntington's disease (HD) (20). In consideration of the hypothalamic area including the suprachiasmatic area, the hypothalamus can be used as a region of interest (ROI).

FC analysis detects abnormalities in brain functional network connectivity at baseline and reflects changes in the interaction and functional coordination among multiple brain regions. However, it is unable to precisely determine sites with abnormal activity. Regional homogeneity (ReHo) analysis of resting-state fMRI reflects the resting-state functional intensity of a certain brain region. Neuroimaging technology is increasingly being harnessed to study circadian rhythms; especially, studying FC and ReHo using resting-state fMRI provides novel approaches to examine the mechanisms of circadian rhythm disorders. The combination of the two methods can better explain and reflect the state of brain function. Previous studies have used the ReHo method to analyze the brain characteristics of normal individuals and demonstrated the existence of cerebral laterality in the resting state and lateralization differences in functional tasks (21, 22). In addition, studies have reported sex-based differences in ReHo in normal individuals and changes in ReHo value of the ipsilateral motor cortex during movement, although changes in movement frequency have minimal effects on the BOLD signal (23). It is suggested that sex may influence the analysis of brain function. According to the international classification of sleep disorders (ICSD-3), circadian rhythm sleep disorders (CRSDs) can be divided into two categories based on underlying mechanisms: intrinsic CRSDs, which comprise diseases characterized by changes in endogenous oscillators (24); and extrinsic CRSDs, which involve external environment and endogenous circadian clock misalignment. The latter category includes shift-work sleep disorder (SWSD) and jet lag. Female night shift nurses in the hospital wake up at night and sleep during the day, which opposes the normal 24-h circadian rhythm; this population constitutes a typical group with CRSDs. In this study, we evaluated the sleep habits of female night shift and female day work nurses using the Morningness-Eveningness Questionnaire (MEQ) (25). Sleep quality and mood were assessed using questionnaires, and blood levels of melatonin and cortisol were collected at five time points. Further, fMRI examinations of participants in both groups were performed. We hypothesized that circadian rhythm disorders would lead to sleep disorders, abnormal melatonin and cortisol rhythms, and corresponding sleep-related functional brain changes in night shift workers. The purpose of this study was to evaluate changes in whole-brain ReHo and hypothalamus-seeded FC associated with circadian rhythm disorders using resting-state fMRI and to elucidate the relationships of these parameters with sleep disorders in night shift nurses.

## Materials and Methods

This study was conducted at the Neurorehabilitation Department of the China Rehabilitation Research Centre between March 2019 and May 2019. All research procedures were approved by the Ethical Committee of China Rehabilitation Research Centre and were conducted in accordance with the Declaration of Helsinki (CRRC-IEC-RF-SC-005-01). Both healthy night shift and day work nurses were recruited from the population of full-time female nurses working at the facility using advertisements.

### Inclusion Criteria

The inclusion criteria for day work nurses were (1) female, 18–35 years old; (2) no night shift history; (3) no sleep disorder for at least half a year before participation; (4) regular work and rest, not staying up later than 23 p.m. (UTC + 8); (5) healthy without other nervous system diseases; (6) not taking anxiety and depression drugs and sleep-regulating medications; (7) informed consent.

For night shift nurses the inclusion criteria were (1) female, 18–35 years old; (2) long term regular night shift for at least half a year, obvious day rest, and at least 6 h of night work; (3) no other nervous system disorders; (4) not taking anxiolytics, antidepressants, or sleep-regulating medications; (5) informed consent.

### Exclusion Criteria

For all participants the exclusion criteria were (1) lack of informed consent; (2) taking hypnotic drugs; (3) mental disorders such as anxiety or depression; (4) failure to cooperate with the completion of the MRI examination, or intracranial abnormalities of MRI.

### Participants

All participants provided written informed consent for their enrolment in the study. Participants completed general intake information forms and questionnaire surveys, allowed blood sample collection, and underwent MRI examination. None of the participants had a history of smoking or drinking, hypertension, diabetes, hyperlipidemia, sleep apnea syndrome, cardiovascular or cerebrovascular disease, fatty liver, or atrial fibrillation. None of the participants were using contraceptives. Height, weight, and body mass index (BMI) were recorded. None of the participants had traveled across time zones or had been administered hypnotics in the 6 months prior to study participation. Participants in the night shift nurses' group were all scheduled for duty for 12 h during the night, 24 h off, and then had another 12-h night shift. All participants in this group had between 1and 8 years of night shift work experience (mean, 4.22 years). For 7 days prior to laboratory admission, the 9 day-work nurses maintained their daily routines and slept for 8 h at regular times each night under dim light conditions (<10 lux) at home.

### Questionnaire Survey

Participants completed a questionnaire survey. A general information questionnaire was used to obtain information on age, education, and other characteristics; evening or morning chronotype was assessed using MEQ (25), and symptoms of depression were measured using Beck Depression Inventory (BDI) (26). Furthermore, sleep quality was assessed among all participants using Pittsburgh Sleep Quality Index (PSQI) (27), and daytime somnolence was measured using Epworth Sleepiness Scale (ESS) (28).

### Measurement of Body Mass Index

The participants stood upright, looked straight ahead, and breathed calmly when their height and weight were measured. BMI (kg/m^2^) is calculated as a person's weight in kilograms divided by the square of their height in meters. According to the WHO standard, participants were divided into the following five groups based on their BMI: underweight, <18.5 kg/m^2^; normal weight, 18.5–24.9 kg/m^2^; overweight, 25.0–29.9 kg/m^2^; obese, 30.0–34.9 kg/m^2^; severely obese, >35 kg/m^2^.

### Blood Sample Collection

Participants were scheduled to enter individual quiet rooms in the neurorehabilitation department at 20:00 (UTC + 8) and remain there until 18:00 (UTC + 8) the next day. Food and water were freely available. Light intensity in the room was set at 100 lux during the time of wakefulness and at <10 lux during sleep time. The ambient temperature was maintained at 23 ± 1°C throughout the study. Peripheral venous blood was collected five times a day at 22:00, 2:00, 6:00, 10:00, and 16:00 (UTC + 8). Peripheral venous blood collection at 2:00 (UTC + 8) was performed under minimal (~10 lux) light intensity. All individuals reported that they fell asleep without insomnia after the blood was drawn. Samples were maintained at room temperature (22–25°C) for 30 min. After centrifugation (5,000 × g for 10 min), serum was collected and cryopreserved at −80°C until subsequent use.

### Detection of Serum Melatonin and Cortisol Levels

Plasma melatonin concentrations were measured using the Human Melatonin (MT) ELISA Kit (batch No: L05015328, Wuhan CUSABIO) according to the manufacturer's instructions. Serum cortisol concentrations were measured using a Human Cortisol ELISA kit (batch No: 1012019, Shanghai MiBio) according to the manufacturer's instructions. Experimenters were blinded to groups and general participant information. The optical density (OD) was measured at 450 nm using an enzyme-labeling instrument within 10 min. A standard curve was constructed according to the concentration and OD value of the standard product. The sample concentration was then calculated according to the standard curve equation.

### Neuroimaging

After blood samples were collected at 16:00 the next day (UTC + 8), fMRI was performed at 17:00 (UTC + 8). Imaging data were acquired using a Philips Achieva 3T MRI scanner with an 8-channel head coil while participants were in a conscious state. Whole-brain coverage gradient echo-planar imaging data were acquired parallel to the AC-PC line with the following parameters: 13 min 19 s, TR = 2,000 ms, TE = 30 ms, flip angle = 90°, voxels = 3 × 3 × 3 mm^3^, number of slices = 50, gap = 0.6 mm, FOV = 220 × 220 mm, and matrix = 80 × 80. Standard high-resolution 3D anatomical T1-weighted scans [sagittal acquisition, repetition time (TR) = 8.2 ms, echo time (TE) = 3.2 ms, flip angle = 8°, isotropic voxel = 1 mm, number of slices = 301, matrix = 256 × 256] were collected to facilitate co-registration. All patients were scanned by the same radiologist.

### Neuroimaging Preprocessing

Preprocessing and analysis of fMRI data were performed using DPABI [Data Processing & Analysis for (Resting-State) Brain Imaging] (29). The regional mean value of quantitative calculation was extracted based on an anatomical automatic labeling (AAL) map (30). Digital Imaging and Communications in Medicine (DICOM) data collected by the device were converted into an analyzable NIFTI format. After assessing the data, time slice and head movement corrections were performed. The EPI template in Montreal Neurological Institute (MNI) space was used as the reference standard. The average image obtained after head movement correction was used as the source image to estimate the registration parameters. MRIs were spatially normalized into the T1MRI template of standard MNI using a 12-parameter affinity transformation and non-linear normalization by a 7 × 8 × 7 basis function. Finally, all data were convolved with a Gaussian kernel filter of 8 × 8 × 8 mm^3^ full-width half maximum (FWHM) to improve the normality of data distribution and to compensate for inexact spatial normalization. As signals related to physiological activity are concentrated in the low-frequency band, the data were filtered with a low-frequency filter of 0.01–0.1 Hz prior to statistical analysis.

### Neuroimaging Analysis

As mentioned in the introduction, the hypothalamus was selected as the seed area for FC analysis. Voxel-by-voxel analysis revealed no significant difference between the day work and night shift groups; therefore, the mean seed-based FC of the hypothalamus and mean whole-brain ReHo were compared between groups with independent sample *t*-test using the AAL map as the source of ROI. Subsequently, we investigated the association of group differences of mean seed-based FC or mean whole-brain ReHo with cortisol or melatonin levels using Spearman correlation analysis. The associations of PSQI and ESS scores were also analyzed with the mean FC and mean brain ReHo using the same test. The significance threshold of brain regions with statistically significant differences was determined as *P* < 0.05.

### Statistical Analysis

Clinical parameters are presented as means and standard deviations. Continuous variables are presented as medians and first and third quartiles. The PSQI, ESS, and BDI scores, which were normally distributed, were analyzed using analysis of variance (ANOVA) and single-sample K-S test. Serum melatonin and cortisol levels were compared using an independent sample *t*-test between the two groups. Pearson or Spearman correlation analysis of PSQI score, ESS score, years of shift work, serum melatonin level, and cortisol level were identified as significantly different variables. Normally and non-normally distributed data were analyzed using Pearson correlation analysis and Spearman correlation analysis, respectively. All *P*-values were two-tailed. Statistical significance was set at a value of *P* < 0.05.

## Results

### Demographic Characteristics of the Study Population

Nine healthy female night shift nurses aged 22–33 years (mean ± standard deviation, 27.33 ± 4.243) and nine healthy female day work nurses aged 21–36 years (mean ± standard deviation, 27.22 ± 5.167) participated in this study. All participants in the night shift group had 1–8 years of night shift work experience (mean ± standard deviation, 4.222 ± 2.224). Five-night shift nurses reported poor subjective sleep, whereas similar complaints were not reported by day work nurses. No significant difference was observed in BMI between the two groups (*P* = 0.276).

### Questionnaire Analysis

BDI scores did not differ significantly between the two groups. Based on the MEQ scores, 12 of the participants were classified as the intermediate type; the remaining six participants were classified as the morning type (Table 1). PSQI and ESS scores of night shift nurses were significantly higher than those of day work nurses, indicating poorer sleep quality and more daytime sleepiness (*P* = 0.002 and *P* = 0.004, respectively). Pearson correlation analysis revealed positive correlations of years of shift work with PSQI (*r* = 0.706, *P* = 0.033) and ESS scores (*r* = 0. 674, *P* = 0.046). A positive correlation was also observed between PSQI score and BMI (*r* = 0.484, *P* = 0.042).

**Table 1:** General information and questionnaire responses.

	**Night shift nurses**	**Day work nurses**	***P*-value**
*N*	9	9	
Age (years)	27.33 ± 4.243	27.22 ± 5.167	0.961
Body mass index (BMI)	21.767 ± 3.310	20.033 ± 3.204	0.276
MEQ	58.89 ± 12.444	53.11 ± 5.349	0.219
Morning type	4	2	0.317
Night type	0	0	
Intermediate type	5	7	
BDI	9.78 ± 9.628	9.33 ± 10.84	0.928
ESS	8.78 ± 3.456	4 ± 2.449	0.004
PSQI	8.44 ± 3.9	3.11 ± 1.900	0.002

*BMI, body mass index; PSQI, Pittsburgh sleep quality index; BDI, beck depression inventory; ESS, Epworth sleepiness scale; MEQ, morningness-eveningness questionnaire*.

### Blood Sample Analysis

**Figure 1:**
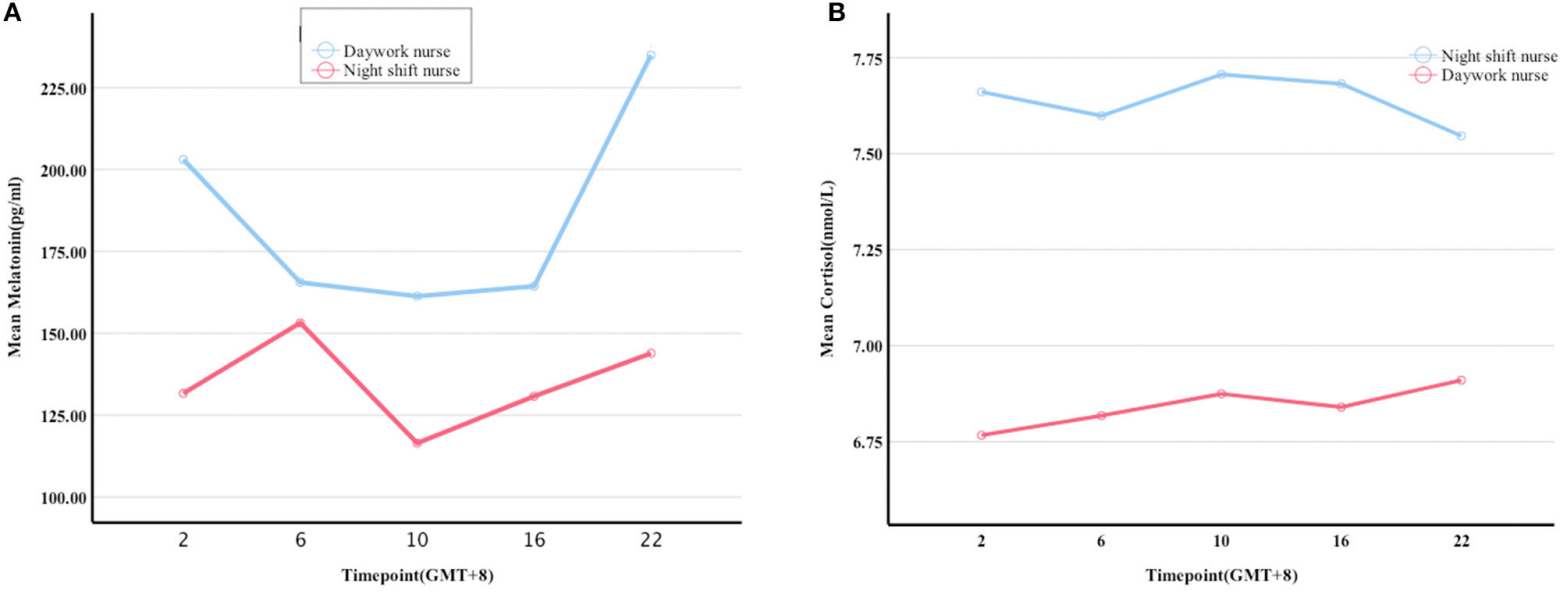
Melatonin and cortisol levels at five timepoints within a 24 h period. **(A,B)** differences in the levels of melatonin and cortisol between the night shift and day work nurses at 2:00, 6:00, 10:00, 16:00, and 22:00 (UTC + 8), respectively.

Melatonin levels were significantly lower in night shift nurses than in day work nurses (*P* = 0.006, Figure 1B). A trend toward higher cortisol levels in night shift nurses than in day shift nurses was also observed, but the difference did not reach statistical significance (*P* = 0.06; Figure 1A). The differences in melatonin and cortisol levels at the five timepoints were further analyzed (UTC + 8). No significant between-group differences were observed in cortisol levels at any time point. Melatonin levels were lower in night shift nurses than in day work nurses at 22:00 (UTC + 8) (Table 2). Cortisol levels in both groups failed to follow a circadian rhythm. No significant effect of age on melatonin secretion was observed. There was a negative association between melatonin secretion and the number of years of shift work, predominantly at 2:00 (*r* = −0.787, *P* = 0. 012) and 16:00 (*r* = −0.717, *P* = 0.003). Cortisol secretion increased with age (*P* < 0.05), but no significant effect of the number of years of shift work on cortisol secretion was noted. There was no significant correlation between the secretion of melatonin and cortisol or BMI.

**Table 2:** Results of blood sample analysis.

	**Night shift nurses**	**Day work nurses**	***P*-value**
*N*	9	9	
Cortisol (nmol/L)	7.639 ± 1.745	6.842 ± 2.203	0.060
Melatonin (pg/mL)	135.179 ± 72.504	185.832 ± 97.596	0.006
Melatonin (pg/mL) (2:00)	131.638 ± 79.141	203.048 ± 97.320	0.107
Melatonin (pg/mL) (6:00)	153.169 ± 89.659	165.514 ± 101.611	0.788
Melatonin (pg/mL) (10:00)	116.433 ± 79.587	161.308 ± 97.889	0.302
Melatonin (pg/mL) (16:00)	130.772 ± 58.586	164.359 ± 69.621	0.285
Melatonin (pg/mL) (22:00)	143.881 ± 62.955	234.930 ± 116.005	0.055

### Functional MRI Analysis

#### Changes in Resting-State Whole-Brain ReHo

**Figure 2:**
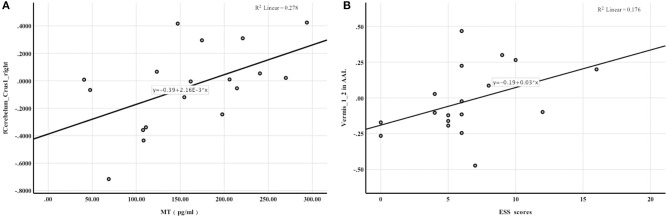
Spearman rank correlation analysis. **(A)** the activation of Cerebelum_Crus1_right in AAL was positively correlated with melatonin levels; **(B)** FC enhancement between the hypothalamus and Vermis_1_2 in AAL was associated with higher ESS scores.

Mean whole-brain ReHo analysis of the two groups revealed a decrease in the activation of the bilateral cerebellar hemispheres and an increase in the activation of the bilateral superior occipital gyrus and left superior parietal gyrus in night-shift nurses (Table 3). Spearman's correlation analysis revealed that higher PSQI (*r* = −0.529, *P* = 0.024) and ESS scores (*r* = −0.716, *P* = 0.001) were associated with weaker activation of the right cerebellar hemisphere. Greater activation of the left superior parietal gyrus was correlated with higher PSQI (*r* = 0.539, *P* = 0.021) and ESS scores (*r* = 0.582, *P* = 0.011). Additionally, greater activation of the right superior occipital gyrus was correlated with higher PSQI (*r* = 0.522, *P* = 0.026) and ESS scores (*r* = 0.661, *P* = 0.001). Further correlation analysis revealed that a decrease in melatonin level was significantly correlated with a decrease in cerebellar hemisphere activation (*r* = 0.583, *P* = 0.011, Figure 2A). No correlations of ReHo brain changes with cortisol levels or BDI scores were identified.

**Table 3:** Differences in ReHo area in the resting state between night shift and day work nurses.

**Location in AAL map (19)**	**Brain area**	**Mean ± SD**	**Mean ± SD**	***T***	***P*-value**
Cerebelum_Crus1_left	Left cerebellar hemisphere	−0.0544 ± 0.1329	0.1681 ± 0.2798	−2.156	0.047
Cerebelum_Crus1_right	Right cerebellar hemisphere	−0.2372 ± 0.2506	0.1555 ± 0.2078	−3.620	0.002
Occipital_Sup_left	Left superior occipital gyrus	0.8023 ± 0.2199	0.5333 ± 0.2945	2.195	0.043
Occipital_Sup_right	Right superior occipital gyrus	0.9401 ± 0.1996	0.5784 ± 0.3896	2.480	0.025
Parietal_Sup_left	Left Superior parietal gyrus	0.4253 ± 0.1934	0.1385 ± 0.1920	3.157	0.006

#### Changes in Resting-State FC of the Hypothalamus as the Seed Region

Significant between-group differences were observed in the right middle cingulate gyrus, right putamen, and vermis (Table 4), suggesting that FC between the hypothalamus and right middle cingulate gyrus, right putamen, and cerebellar vermis was more enhanced in night shift nurses than in day work nurses. No correlations of FC with PSQI scores and melatonin levels were noted. FC enhancement between the hypothalamus and vermis was associated with higher ESS scores (*r* = 0.501, *P* = 0.034, Figure 2B). No significant correlations of FC changes with cortisol levels and BDI scores were identified.

**Table 4:** Differences in the resting-state FC area between night shift and day work nurses.

**Location in AAL (19) map**	**Brain area**	**Mean ± SD**	**Mean ± SD**	***T***	***P*-value**
Cingulum_Mid_right	Median cingulate and paracingulate gyri	−0.0271 ± 0.1692	−0.1726 ± 0.1136	2.141	0.048
Putamen_right	Right putamen	0.0511 ± 0.1059	−0.0920 ± 0.1345	2.309	0.023
Vermis_1_2	Cerebellar vermis	0.0930 ± 0.2325	−0.1374 ± 0.1917	2.295	0.036
Vermis_3	Cerebellar vermis	0.0692 ± 0.1768	−0.0972 ± 0.1398	2.216	0.042

## Discussion

**Figure 3:**
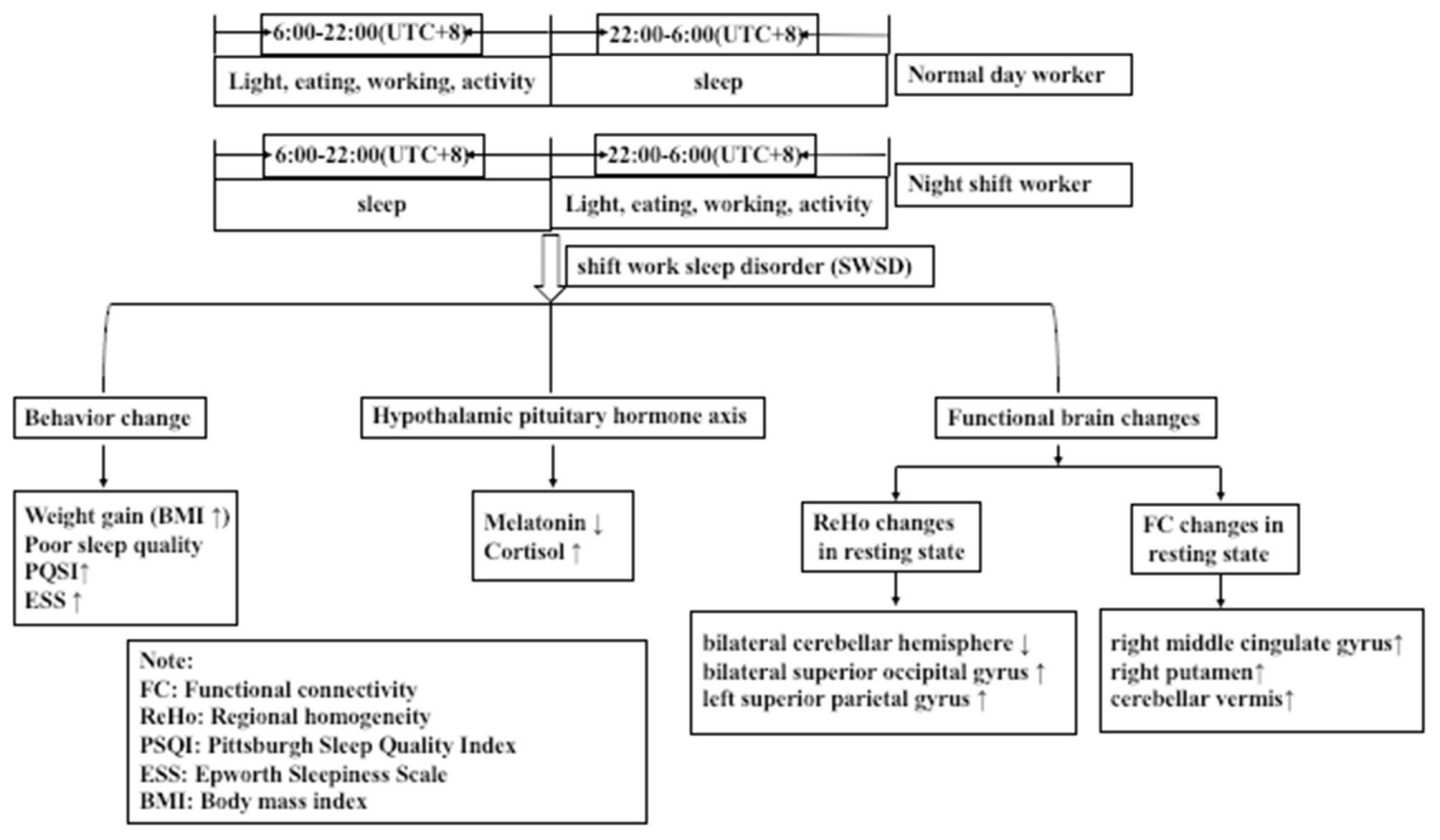
Night shift nurses exhibited greater sleep disorders, hormonal disorders, and changes in the activation of functional brain areas than did day work nurses.

To the best of our knowledge, this is the first study to assess night shift nurses as the research subject to confirm the presence of circadian rhythm disorder from the aspect of hormone levels, and to identify that circadian rhythm disorder can lead to changes in brain function. We examined sleep quality and daytime somnolence in night shift nurses using sleep questionnaires and measurements of serum melatonin levels. Further, we analyzed changes in whole-brain ReHo and FC to the hypothalamus using resting-state fMRI. We observed that sleep quality decreased, daytime somnolence increased, and melatonin levels were reduced in night shift nurses. Further, we noted decreased activation of the bilateral cerebellar hemisphere and increased activation of the bilateral superior occipital gyrus and left superior parietal gyrus in night shift workers. Decreased activation of the right cerebellar hemisphere was associated with decreased melatonin secretion, decreased sleep quality, and increased daytime somnolence. Considering that the suprachiasmatic nucleus is located in the hypothalamus, we selected the hypothalamus as the seed area for FC analysis and observed that FC to the right middle cingulate gyrus, right putamen, and cerebellar vermis was enhanced; in particular, the enhancement of FC between the hypothalamus and cerebellar vermis was related to the increase in daytime somnolence (Figure 3). Despite its small sample size, this is the first study to examine changes in the brain function of otherwise healthy individuals with circadian rhythm disorders.

Our results demonstrate that night shift work resulted in sleep disorders; the longer the experience of night shift work, the poorer the sleep quality and the higher the daytime somnolence, which is consistent with previous findings (8). Meanwhile, the poorer the sleep quality, with higher the BMI. Possible mechanisms for this association include anxiety or eating at night, resting during the day, decreased exercise, or stress. In addition, because of the small sample size, no significant difference in circadian phenotype between the two groups was detected. To explore whether circadian phenotype has an impact on the brain function of participants with circadian rhythm disorder, a larger sample size is needed, and a more in-depth study on circadian rhythm disorder must be conducted according to the “owls” and “larks” types of the MEQ scale.

Epidemiological studies have demonstrated that shift work decreases melatonin secretion, induces abnormal rhythms of cortisol secretion, and disrupts inflammatory responses (3–5). In this study, night shift nurses exhibited a typical daytime reversal and served as a typical circadian rhythm population. Analyses revealed that melatonin levels were significantly decreased at night and cortisol rhythms were perturbed, consistent with previous findings. We also observed that the longer the experience of shift work, the greater the reversal of melatonin rhythm. With an increase in age, the secretion of cortisol increased. However, we did not observe an association between increased time engaged in night-shift work and cortisol levels, nor was there a significant correlation between BMI and cortisol levels, which is inconsistent with previous findings (31–34). Our failure to detect an association may be related to the small sample size.

Notably, the overall cortisol secretion rhythms of control participants in this study differed from normal rhythms, possibly due to the sample collection method. In our study, blood sample collection was conducted at five timepoints, which may have contributed to an increase in cortisol levels during collection. Melatonin or cortisol levels can be analyzed using blood, urine, hair, feces, or saliva samples. Most studies on melatonin or cortisol used urine or saliva samples (4, 5, 35), and evaluation of salivary samples is preferred to blood samples because venipuncture is associated with elevated cortisol levels (36). This may also partly explain the disruption of cortisol rhythms in day work nurses. Peripheral blood collection methods used in other studies typically involved placement of an intravenous catheter for blood sampling in the forearm vein of participants to avoid stress and to minimize impact on sleep (37). This approach should be adopted in the future to improve blood sample collection methods.

The FC of human brain networks underpins cognitive function (38, 39). Sleep and circadian rhythm disorders may interfere with cognitive processes, and perturbations of FC are associated with sleep and nervous system diseases. Facer-Childs et al. categorized early and late circadian phenotypes using the Munich Chronotype Questionnaire. Those authors selected the DMN as the seed area, which is easily affected by sleep onset and sleep deprivation. Their results indicated fundamental differences in the DMN between early and late circadian phenotypes. Resting-state FC of the DMN of late circadian phenotypes displayed dysfunction, that is decreased attention and increased subjective sleepiness (9). Indeed, sleep disorders and circadian rhythm disorders can lead to changes in brain FC. Circadian rhythm and homeostasis affect resting-state FC but are not affected by circadian typology (40). Changes in resting-state brain FC have been reported in various nervous system diseases with circadian rhythm disorders, such as delirium, Huntington's disease, and bipolar disorder (15, 20, 41). Park et al. investigated the regional cerebral blood flow in shift workers using perfusion MRI (pMRI) and observed that it decreased significantly in the cuneus, fusiform/ parahippocampal gyri, and cerebellum of the right hemisphere, while it was increased in the inferior occipital gyrus of the left hemisphere. Moreover, perfusion changes in shift workers were significantly correlated with depression and insomnia severity (8). These studies support the notion that circadian rhythm disorders affect functional brain change. McGlashan et al. reported a positive correlation between BOLD fMRI activation in the human suprachiasmatic area in response to light and melatonin suppression (9). Our results revealed a difference in mean brain ReHo between night shift and day shift nurses, and poorer sleep quality was related to weaker activation of the right cerebellum, and greater activation of the left superior parietal gyrus and right superior occipital gyrus. Moreover, the decrease in melatonin level was associated with the decrease in sleep quality as well as the decrease in the activation of the right cerebellum. The activation of the right cerebellum decreased after circadian rhythm disorder in night shift nurses, which was related to poor sleep quality, and may be involved in melatonin pathway mediated sleep regulation. Furthermore, resting-state FC also suggested that the FC between the cerebellar vermis and the hypothalamus was enhanced and this enhancement was associated with an increase in daytime sleepiness. Most studies suggest that the cerebellum may also be involved in the regulation of the sleep-wake cycle. For example, the most significant pathological change in patients with spinocerebellar ataxia is extensive degeneration of the cerebellum, and these patients often experience daytime sleepiness (42, 43). In addition, after bilateral cerebellar peduncles were damaged in cats, their awakening time was significantly reduced, and their drowsiness increased (44). Further, patients with chronic insomnia, fatal familial insomnia, obstructive sleep apnea, and daytime sleepiness exhibit a decrease in cerebellar volume (43, 45). This suggests that the cerebellum participates in the regulation of the sleep-wake cycle. Indeed, the cerebellum has been proposed to project to functional sleep areas of the thalamus and hypothalamus (46–48). Additionally, deep cerebellar nuclei form synaptic connections with multiple brain regions involved in arousal initiation and/or maintenance, such as the ventral thalamus, which are thought to promote the conversion from sleep to arousal (49, 50). Collectively, these findings suggest that activation of the cerebellum is related to sleep disorders. Combined with this, our findings may suggest that circadian rhythm disorder leads to cerebellar dysfunction and that the FC between the cerebellum and thalamus is enhanced. In our FC analysis, we did not observe any correlation between FC change with melatonin level or PSQI score, but a positive correlation was observed between enhanced cerebellar vermis FC and ESS scores, suggesting that enhanced FC between the hypothalamus and cerebellar vermis was associated with daytime subjective sleepiness. However, it is not clear whether this suggests that the enhancement of FC between the thalamus and cerebellum is related to compensatory sleep during the daytime. In order to further elucidate this issue, a larger sample size is needed. In addition, future research can combine EEG and fMRI to detect differences in the sleep-related brain regions between participants with circadian rhythm disorders and healthy participants to determine the functional changes in these different brain areas across the sleep-wake cycle using a larger population.

In our study, night shift nurses displayed a trend of higher cortisol level and weight gain than did day shift nurses, but did not differ significantly in terms of depression; furthermore, no obvious functional brain change was observed with depression in the former group. The possible reason is that the BDI score is not sensitive to early symptoms and cannot assess anxiety and other emotional disorders. Another explanation for the inconsistency between our findings and previous research is the small sample size. Recent studies have demonstrated that sleep influences plasticity in the visual cortex. Sleep deprivation reduced task-related activity in the frontal and parietal regions as well as visuospatial attention-related and task-related activity (51). Our results indicated greater activation of ReHo in the right superior occipital gyrus and the left superior parietal gyrus. Compensatory mechanisms such as subjective effort may partially improve the level of attention and maintain certain operational ability. In this regard, compensatory efforts may have occurred in relation to the increase in ReHo in the left superior parietal gyrus and right superior occipital gyrus in the night shift nurses.

The basal forebrain is implicated in various bodily functions, including awakening, circadian rhythm, water drinking, body fluid balance, and feeding (52, 53). The putamen, a component of the basal forebrain connected to the thalamus and hypothalamus, regulates sleep and wakefulness. Decreased putamen gray matter volume, as well as a negative correlation between putamen atrophy and arousal index, has previously been reported in patients with insomnia (8). Therefore, increased connectivity between the putamen and hypothalamus may be implicated in the regulation of sleep-wake disorders. The cingulate gyrus is a component of the limbic system; the hypothalamus is connected to the cingulate gyrus via projection fibers of the papillary body and has an integrated role in the regulation of behavior, cognition, and emotion. Lesions of the cingulate gyrus result in various symptoms, such as inattention, cognitive dysfunction, and apathy. Cingulate gyrus dysfunction is noted in patients with sleep deprivation or delirium (7, 10, 54). In this study, we observed that FC between the hypothalamus and putamen, right cingulate gyrus was enhanced. Night shift nurses had obvious complaints of decreased daytime attention or increased daytime sleepiness. Although no significant difference in BDI scores between the two groups was noted, the increased FC from the hypothalamus to the cingulate gyrus may be a compensatory mechanism to adjust emotional responses accordingly. Some studies have explored changes in brain function after 36 h of sleep deprivation and their correlation with the findings of psychomotor vigilance tests (PVT). The FC between cerebellum and central posterior gyrus decreased, which is negatively correlated with the prolongation of PVT response time; furthermore, the FC between cerebellum and bilateral caudate nucleus was enhanced, which is positively correlated with prolongation of PVT response time, suggesting that the change in cerebellum FC may be related to the impairment of psychomotor vigilance after sleep deprivation. The cerebellum is not only involved in sleep regulation, but also in cognitive functions such as responsiveness and alertness (55). Another study was conducted with 22 female patients with acute sleep deprivation. The brain activity related to sleep deprivation was analyzed based on the amplitude of low-frequency fluctuations (ALFF). The results indicated that attention decreased, response time increased, and ALFF of the right anterior cerebellar lobe decreased significantly. This further suggests that the cerebellum may participate in cognitive function and that this cognitive function might be stabilized and enhanced by sleep (56). Canto summarized that learning-related time, procedural memory formation, and spatiotemporal predictions of motor actions, which are known to be controlled at least in part by the cerebellum, are facilitated by sleep (57). Accordingly, these results led to a theoretical model of sleep-dependent memory consolidation, and the possible mechanism of memory consolidation related to the cerebellum during sleep (57). In future research, it is important to study the correlation of abnormal functional activation in brain areas associated with emotion, cognition, and sleep in a large sample of participants with circadian rhythm disorders.

Although we recruited nurses from the same work environment and same department to exclude differences in sex and levels of work stress, to the best of our ability, potential differences that may be present among our participants remain the main limitations of our study. Additional limitations include the inclusion of only female participants and small sample size. As such, the results may not be fully generalizable to males. Furthermore, significant differences in attention and emotion may be detected in a larger sample.

In conclusion, dysrhythmia of circadian rhythms contributes to resting-state functional changes in the cerebellum. In severe cases, this may be accompanied by functional changes in attention and emotion-related brain regions. In addition, for this type of population with circadian rhythm disorder, further investigation into whether transcranial magnetic stimulation can effectively regulate the abnormal activation area is warranted.

## Data Availability Statement

The original contributions presented in the study are included in the article/supplementary material, further inquiries can be directed to the corresponding author/s.

## Ethics Statement

The studies involving human participants were reviewed and approved by the Ethical Committee of China Rehabilitation Research Centre and were conducted in accordance with the Declaration of Helsinki (CRRC-IEC-RF-SC-005-01). The patients/participants provided their written informed consent to participate in this study.

## Author Contributions

XW was the study coordinator and contributed to the development of the study idea, analysis of demographic data and results of questionnaires and actigraphy, discussion of analysis and results, and writing and revision of the manuscript. YW contributed to the scripting of data preprocessing, as well as writing and revision of the manuscript. FB contributed to fMRI data acquisition, blood sample analysis, and revision of the manuscript. HL, YC, and LZ contributed to the collection of blood samples, analysis of demographic data and questionnaire results, and revision of the manuscript. LL contributed to the revision of the manuscript. TZ contributed to the study idea, the discussion of analysis and results, and revision of the manuscript. All authors contributed to the article and approved the submitted version.

## Conflict of Interest

The authors declare that the research was conducted in the absence of any commercial or financial relationships that could be construed as a potential conflict of interest.
